# Regular Intake of Pistachio Mitigates the Deleterious Effects of a High Fat-Diet in the Brain of Obese Mice

**DOI:** 10.3390/antiox9040317

**Published:** 2020-04-15

**Authors:** Domenico Nuzzo, Giacoma Galizzi, Antonella Amato, Simona Terzo, Pasquale Picone, Laura Cristaldi, Flavia Mulè, Marta Di Carlo

**Affiliations:** 1 Istituto per la Ricerca e l’Innovazione Biomedica (IRIB), CNR, Via Ugo La Malfa 153, 90146 Palermo, Italy; domenico.nuzzo@irib.cnr.it (D.N.); giacoma.galizzi@irib.cnr.it (G.G.); pasquale.picone@irib.cnr.it (P.P.); laura.cristaldi@irib.cnr.it (L.C.); 2 Dipartimento di Scienze e Tecnologie Biologiche Chimiche e Farmaceutiche (STEBICEF), Università degli Studi di Palermo, viale delle Scienze, 90128 Palermo, Italy; antonella.amato@unipa.it (A.A.); simona.terzo01@unipa.it (S.T.); flavia.mule@unipa.it (F.M.); 3 Dipartimento di Neuroscienze e Biologia Cellulare, Università di Palermo, Via del Vespro 129, 90127 Palermo, Italy

**Keywords:** pistachio, HFD, obesity, oxidative stress, neurodegeneration

## Abstract

Obesity has been associated with neurodegeneration and cognitive dysfunctions. Recent data showed that pistachio consumption is able to prevent and ameliorate dyslipidemia, hepatic steatosis, systemic and adipose tissue inflammation in mice fed a high-fat diet (HFD). The present study investigated the neuroprotective effects of pistachio intake in HFD mice. Three groups of mice were fed a standard diet (STD), HFD, or HFD supplemented with pistachio (HFD-P) for 16 weeks. Metabolic parameters (oxidative stress, apoptosis, and mitochondrial dysfunction) were analyzed by using specific assays and biomarkers. The pistachio diet significantly reduced the serum levels of triglycerides and cholesterol in the HFD model. No difference was observed in the index of insulin resistance between HFD and HFD-P. A higher number of fragmented nuclei were found in HFD cerebral cortex compared to STD and HFD-P. A decrease in reactive oxygen species, singlet oxygen and phosphorylated extracellular signal-regulated kinase, and an increase of superoxide dismutase 2 and heme oxygenase expression were found in the brains of the HFD-P samples compared to HFD. Furthermore, the impaired mitochondrial function found in HFD brain was partially recovered in HFD-P mice. These results suggest that the regular intake of pistachio may be useful in preventing obesity-related neurodegeneration, being able to reduce both metabolic and cellular dysfunctions.

## 1. Introduction

In the last 50 years, the prevalence of neurodegenerative diseases, including different forms of dementia, is increasing, becoming a social and economic burden. Recent evidence indicates that metabolic dysfunctions may play a key role in the development of neurodegeneration [1,2].

It is well known that a high-fat diet (HFD) can lead to obesity, type 2 diabetes, non-alcoholic fatty liver disease, and neurodegenerative diseases [1,2,3,4,5]. A correct lifestyle, combining a healthy diet with regular physical exercise, could prevent metabolic dysfunctions and consequently protect from the related-neurodegenerative disorders.

Natural remedies are currently drawing attention as protective agents in treating obesity-related dysfunctions [6,7]. Food with antioxidant, anti-hyperlipidemia, and anti-inflammatory properties could help to reduce the risk of metabolic dysfunctions [8].

The positive effects of the Mediterranean diet on health have been well documented [9,10]. The longevity of one Mediterranean population, over 90 years old without dementia, was attributed to the nutraceutical components of the Mediterranean diet [11]. Functional food has been reported to delay or inhibit neurodegeneration, suggesting their employment as an alternative therapeutic strategy for correlated diseases [12,13,14].

Benefits of nut consumption (mainly pistachios, walnuts, and almonds) have been described in studies on both animals and humans [15,16]. Daily nut consumption can improve dysmetabolic conditions such as obesity, type 2 diabetes, and related cardiovascular diseases [17,18]. In particular, *Pistacia atlantica* oleoresin has been proposed as an agent that protects the body against conditions associated with oxidative stress [19], including memory impairment, in lipopolysaccharide-treated rats [20]. Nevertheless, the potential beneficial impact of nut intake on neurodegenerative disorders, as well as on other cognitive-behavioral deficits, has been poorly explored.

Compared to other nuts, pistachios possess a healthier nutritional profile, with low-fat content, high content of polyunsaturated fatty acids (13.3 g/100 g) and mono-unsaturated fatty acids (24.5 g/100 g), minerals (potassium, phosphorus, magnesium, and calcium) and vitamins (vitamin A, vitamin E, vitamin C, and vitamins B). Phytochemicals of pistachio show high bioavailability, contributing to the beneficial relationship between pistachio consumption and health-related outcomes [21]. Furthermore, recent data have demonstrated the ability of pistachio consumption in preventing and ameliorating some obesity-related dysfunctions such as dyslipidemia, hepatic steatosis, and systemic and adipose tissue inflammation [15,22]. Accumulation of several lipids associated with an increase in oxidative stress has also been reported in the brain of HFD-fed rodents [23]. Lipid dysmetabolism can lead to neuronal damage, causing related-obesity neurodegenerative diseases [23,24,25,26]. Therefore, we evaluated whether regular pistachio intake has a positive impact, and it exerts beneficial actions in preventing neurodegeneration induced by HFD in the mouse. For this aim, mice were fed an HFD supplemented with pistachios for 16 weeks, and lipids, oxidative stress, mitochondrial dysfunction, and neurodegeneration were studied in the brain and compared with HFD and standard diet (STD) fed mice.

## 2. Materials and Methods

### 2.1. Animals, Diets and Experimental Design

Animal experiments were performed in accordance with the Italian legislative decree No. 26/2014 and the European directive 2010/63/UE, and were authorized by the Ministry of Health (Rome, Italy; Authorization no. 349/2016-PR). Four-week-old male C57BL/6J (B6) mice, purchased from Harlan Laboratories (San Pietro al Natisone-Udine, Italy) were housed under standard conditions of light (12 h light: 12 h darkness cycle) and temperature (23 ± 1 °C) and relative humidity (55 ± 5%). Food and water were freely available ad libitum.

After one week of acclimatization, the mice were randomly divided into three groups: (a) Mice fed a standard diet (STD, *n* = 8); (b) Mice fed High Fat Diet (HFD, *n* = 8); (c) Mice fed an HFD supplemented with pistachio from Valle del Platani, (AG) Sicily, Italy (HFD-P, *n* = 8). Animals were maintained on each diet for 16 weeks. As previously described [22], the diets supplied were: (1) STD (70% of energy as carbohydrates, 20% protein, and 10% fat; 4RF25, Mucedola, Milan, Italy), (2) HFD (60% of energy as fat, 20% protein, and 20% carbohydrates; PF4215, Mucedola, Milan, Italy), (3) HFD with pistachio (HFD-P; 60% of energy as fat, 20% protein, and 20% carbohydrates; PF4215/C, R&S 34/16, Mucedola, Milan, Italy). HFD-P was custom designed and prepared by Mucedola by substituting 20% of the caloric intake from HFD with pistachio (180 g/kg of HFD). Bodyweight, food intake, and caloric intake were recorded weekly.

At the end of the experimental period, all mice, after fasting overnight, were sacrificed by cervical dislocation. Blood was immediately drawn by cardiac puncture, and plasma was recovered after centrifugation at 3000 rpm at 4 °C for 15 min and stored at −80 °C until analysis. Then the entire aortic tree was perfused with Dulbecco’s phosphate-buffered saline containing 2 mM EDTA. Perfusion was carried out via a cannula introduced into the left ventricle, with incision of the right atrial appendage to permit the outflow of blood. Then, the brains were explanted, washed, weighed, and processed for subsequent analysis. Blood glucose, triglyceride, and cholesterol concentrations were measured by using a glucometer (GlucoMen LX meter, Menarini, Florence, Italy) and Biochemistry Analyzer MultiCare (Biochemical Systems International-Srl, Arezzo, Italy), respectively. Quantification of plasma insulin was carried out by ELISA kit for mouse (Alpco diagnostics, Salem, NH, USA) according to the manufacturer’s instructions and homeostasis model assessment of insulin resistance (HOMA-IR) was calculated.

### 2.2. Brain Tissue Preparation

Explanted brains from STD, HFD, and HFD-P mice were coronally cut in two halves obtaining an anterior and a posterior part. One part was homogenated in ice by using a Dounce, then separated into aliquots (5 or 10 mg) and immediately flash-frozen in liquid nitrogen and stored until required for analysis. The other part was used for the histological analysis. Thus, the half brain was fixed in 4% formalin for 24 h followed by graded ethanol (50%, 70%, 85%, 96%) for 5 min each, then embedded in paraffin overnight and subsequently sectioned (5 μm thick) using a microtome.

### 2.3. Tissue Cholesterol Assay

10 mg of frozen homogenate brain tissue was resuspended in 100 μL of PBS and processed using the Amplex red cholesterol assay kit (Life Technology, Monza, Italy), according to the manufacturer’s instructions. Absorbance was measured by using the GloMax^®^ Discover multimode plate reader (Promega, Italy) at 490 nm. Cholesterol concentrations were evaluated by using a standard curve, according to the manufacturer’s instructions.

### 2.4. Lipid Peroxidation Assay

To detect the concentration of brain lipid peroxidation, 10 mg of frozen homogenate brain tissue was resuspended in 300 μL of malondialdehyde (MDA) lysis buffer, and the lipid peroxidation MDA assay (Sigma-Aldrich, Milan, Italy) was used according to the manufacturer’s instructions. Absorbance was measured at 532 nm by using the GloMax^®^ Discover multimode plate reader. To detect the concentration of plasma lipid peroxidation, 20 µL of plasma was processed as described above.

### 2.5. Detection of Oxidative Levels: DCFH-DA Assay

Reactive oxygen species (ROS) generation in the brain was evaluated by using 2′,7′-dichlorofluorescin diacetate (DCFH-DA; Molecular Probes, Eugene, OR, USA). 5 mg of frozen homogenate brain tissue was resuspended in 5 mL of PBS buffer. After centrifugation, 100 μL of the supernatant was plated and incubated for 5 min with 1 µL of DCFH-DA (1 mM). Oxidation levels were evaluated using the GloMax^®^ Discover system (Promega) at 37 °C at an excitation wavelength of 475 nm and an emission wavelength of 555 nm. To evaluate the presence of ROS in the plasma 1 μL of DCFH-DA (1 mM) was added to 20 µL of plasma and processed as described above.

### 2.6. TUNEL Assay

Apoptosis was evaluated by using the in situ cell death detection kit, TMR red (Roche, Monza, Italy) according to the manufacturer’s instructions. Briefly, after progressive hydration, sections of paraffin-embedded brains (5 μm thick) were incubated with permeabilization solution for 8 min, washed in PBS, and incubated with TUNEL reaction mixture for 60 min at +37 °C in a humidified atmosphere in the dark. After washing in PBS, the slides were incubated with Hoechst 33258 (5 μg/mL) for 20 min and analyzed by using a DHL fluorescent microscope (Leica Microsystems, Heidelberg, Germany) at a magnification of 20×.

### 2.7. Lipid Nile Red Staining

To evaluate the presence of lipids in the brain tissue, the deparaffinized brain sections from STD, HFD, and HFD-P mice were hydrated in graded ethanol for 5 min each. After washing in PBS, the sections were stained by using Nile Red (0.5 μL/mL) (ThermoFisher Scientific, San Jose, CA, USA) at room temperature for 1 h. Samples were analyzed by using a DHL fluorescent microscope. To quantify lipids in the brain tissue, 10 mg of frozen brain homogenate were resuspended in 1 mL of PBS and Nile Red (0.5 μL/mL). The homogenate was incubated at room temperature for 15 min. 2 μL of the solution was spotted onto a nitrocellulose membrane, and the fluorescence was visualized by using the Typhoon FLA 9500 scanner (excitation/emission 552/636 nm). The images were analyzed by ImageQuant TL software (GE Healthcare Life, Marlborough, MA, USA).

### 2.8. Singlet Oxygen (^1^O_2_) Species Generation

10 mg of frozen homogenate brain tissue was resuspended in 1 mL of PBS; the samples were centrifuged at 10,000g at 4 °C for 10 min. 48 μL of the supernatant of each sample was mixed with 50 μL of the reagent buffer and 2 μL of 5 mM SOSG agent (Molecular Probes). 80 μL of each sample mixture was added into a well of 96-well plate (Black Microtiter Plate, Thermo Scientific, Vantaa, Finland) and covered with transparent lid. The fluorescence signal was measured at excitation 488 nm and emission 525 nm by a spectrophotometer GloMax^®^ Discover system.

### 2.9. SOD Activity Levels

10 mg of frozen homogenate brain tissue was resuspended in 1 mL of PBS buffer with protease inhibitors (Amersham Life Science, Munich, Germany). To remove insoluble material, tissue lysates were sonicated on ice (cooled for 30 s and sonicated for 30 s twice at low power output, 20 W). After centrifugation (14,000 rpm, at 4 °C, for 30 min), the supernatant was submitted to the Bradford method for protein quantification. A volume corresponding to 50 μg of total proteins was used for SOD enzymatic activity measurement, by using the SOD assay kit (Sigma-Aldrich) according to the manufacturer’s instructions. Absorbance was measured at 450 nm by using the GloMax^®^ Discover multimode plate reader.

### 2.10. Immunofluorescence Analysis

Coronal brain sections were mounted on slides and deparaffinized in xylene and hydrated in a series of graded ethanol (96%, 85%, 70%, 50%) for 5 min each. The slides were incubated at 4 °C overnight with the primary antibody anti-phosphorylated extracellular signal-regulated kinase (p-ERK; 1:25; Santa Cruz Biotechnology, Heidelberg, Germany) and anti-microtubule-associated protein light chain 3 (LC3; 1:25; Santa Cruz Biotechnology). After washing in PBS, the slides were incubated with anti-rabbit Cy3-conjugate secondary antibodies (1:500; Cell Signaling Technology, Danvers, MA, USA). Nuclear staining was performed using Hoechst 33258 (5 μg/mL) for 20 min. The slides were analyzed by using a DHL fluorescent microscope (Leica Microsystems, Heidelberg, Germany) at a magnification of 20×. p-ERK and LC3 positive fluorescence intensity were measured by using a Leica QFluoro program (Leica Biosystems, Wetzlar, Germany). The immunohistochemical staining was run in triplicates per mouse for each antibody and observed by two independent research in four slides.

### 2.11. Total Protein Extraction and Western Blot

Total proteins were prepared by resuspending 10 mg of frozen homogenate in solubilizing buffer (50 mM Tris-HCl, pH 7.4; 150 mM NaCl, 0.5% Triton X-100, 2 mM PMSF, 1 mM DTT, 0.1% SDS) with protease inhibitor (Amersham, Life Science, Les Ulis, France) and phosphatase inhibitor cocktail II (Sigma-Aldrich, Poole, Dorset, UK). Total proteins were quantified by the Bradford method (Bio-Rad, Segrate, Italy). 50 μg of protein samples were resolved by 12% acrylamide gel and transferred onto a nitrocellulose filter for Western blotting. The filter was incubated with anti-superoxide dismutase 2 (SOD2; 1:500, Santa Cruz Biotechnology), anti-heme oxygenase (H-Oxy; 1:1000, Cell Signaling Technology), anti-heat shock protein 60 (HSP60; 1:500, Cell Signaling Technology), anti-mitochondrial dynamin-like GTPase 1 (OPA1; 1:500, Santa Cruz Biotechnology), anti-dynamin-related protein 1 (DRP1; 1:500, Santa Cruz Biotechnology), anti-mitochondrial fission 1 protein (FIS1; 1:500, Santa Cruz Biotechnology), anti-PTEN-induced kinase 1 (Pink1; 1:500, Santa Cruz Biotechnology), anti-RBR E3 Ubiquitin Protein Ligase (Parkin; 1:500, Santa Cruz Biotechnology), anti-ubiquitin-binding protein p62 (p62; 1:500, Santa Cruz Biotechnology), and anti-β-actin (β-Actin; 1:10,000, Sigma-Aldrich). Primary antibodies were detected using the Odyssey^®^ scanner (LI-COR Biosciences, Lincoln, NE, USA), according to the manufacturer’s instructions, using secondary antibodies (anti-mouse and anti-rabbit) labeled with IR790 and IR680 (1:10,000; Life Technology). Band intensities were analyzed with the Odyssey^®^ CLx imaging system, and expression was adjusted to actin expression. The protein levels were expressed as intensity relative to control.

### 2.12. Isolation of Brain Mitochondria

Cytosol and mitochondria fractions from 10 mg of frozen brain tissue, were prepared using Mitochondrial isolation kit (ThermoFischer, Italy) according to the manufacturer’s instructions using buffers provided by the kit. Briefly, the brain homogenate (10 mg) was resuspended in 200 µL of lysis buffer, centrifuged at 2000 g for 3 min to remove cell debris. The supernatant was centrifuged at 10,000g for 5 min and the mitochondrial pellet was washed twice by centrifugation at 10,000g for 10 min and resuspended in the buffer provided by the kit. An aliquot was used to determine protein concentration (2 μg/μL) by the Bradford method. The amount of mitochondrial protein is usually accepted as a mitochondrial quantity [27], and an equal amount (50 μg) of mitochondrial protein was used for each measurement in all experiments.

### 2.13. Mitochondrial Stress

The presence of superoxide in brain isolated mitochondria was analyzed by fluorescence using the MitoSOX Red reagent (Molecular probes, Paisley, UK). A volume corresponding to 50 µg of mitochondrial proteins was incubated with MitoSOX reagent (5 μM) for 10 min at 37 °C in the dark. At the end of the incubation, the solution was centrifuged at 10,000g for 5 min, and the mitochondrial pellet was resuspended in PBS and analyzed by GloMax^®^ Discover multimode plate reader (Promega, Italy) at the excitation wavelength of 514 nm and to record the emission spectrum in the range 540–640 nm. A dilution of the sample (1:50) was used for microscopic inspection (DHL fluorescent microscope Leica Microsystems, Heidelberg, Germany).

### 2.14. Mitochondrial Swelling

The swelling of brain isolated mitochondria was evaluated according to Chapa-Dubocq et al. [27], by measuring the changes in the absorbance of the mitochondrial suspensions at 540 nm using a GloMax^®^ Discover multimode plate reader (Promega, Italy). A volume corresponding to 50 µg of mitochondrial proteins was incubated with 50 µL of buffer (125 mM KCl, 1 mM MgCl_2_, 5 mM malate, 5 mM glutamate, 1 µM EGTA, and 20 mM Tris base) at pH 7.4. The absorbance was monitored for 5 min at 37 °C at 540 nm, and the mitochondrial swelling was indicated by a decrease in the absorbance at 540 nm.

### 2.15. Statistical Analysis

The results are presented as mean ± SEM. A one-way ANOVA was performed, followed by Dunnett’s post hoc test for analysis of significance. Results with a *p*-value < 0.05 were considered statistically significant.

## 3. Results

### 3.1. Effects of Pistachio Intake on Metabolic Parameters

**Figure 1:**
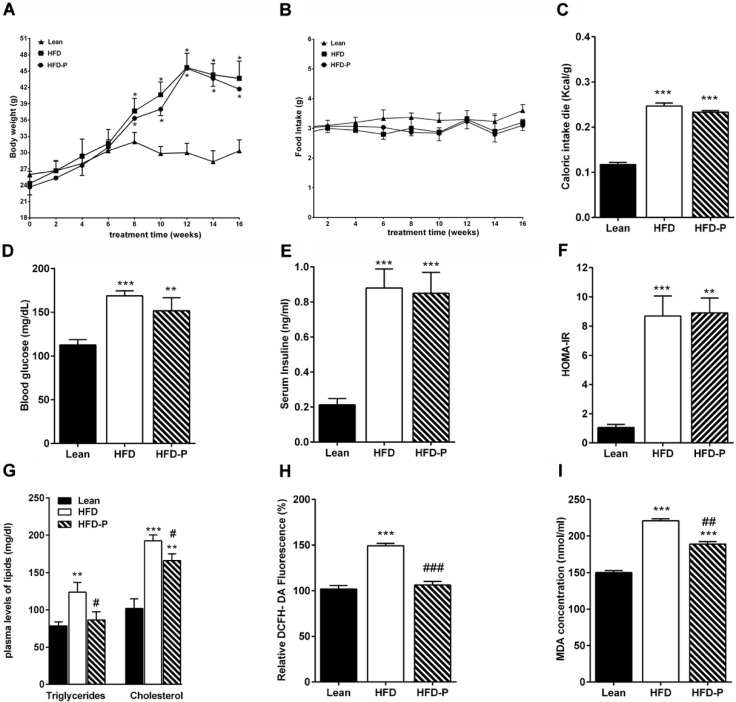
Pistachio consumption attenuates alterations of various metabolic and oxidative parameters in a high-fat diet (HFD) mice. Values in mice after 16 weeks of standard diet (STD), HFD or HFD supplemented with pistachio (HFD-P): (**A**) body weight; (**B**) daily food intake; (**C**) caloric intake; (**D**) fasting glucose concentration; (**E**) serum insulin concentration; (**F**) HOMA-IR; (**G**) plasma levels of triglycerides and cholesterol; (**H**) plasma ROS levels in lean, HFD, or HFD-P mice expressed as % of lean; (**I**) plasma lipid peroxidation levels in the different animal groups. Data are means ± standard error medium (SEM) (*n* = 8/group). * *p* < 0.05, ** *p* < 0.01, *** *p* < 0.001 vs STD. # *p* < 0.05, ## *p* < 0.01, ### *p* < 0.001 vs HFD.

As shown in Figure 1A, HFD and HFD-P mice presented a body weight significantly higher than the lean mice. No difference in the daily food intake was observed among the three different groups (Figure 1B). The net energy intake in HFD and HFD-P animals was higher than lean mice (Figure 1C). Systemic metabolic parameter analysis showed that HFD and HFD-P fasting glycemia, insulin concentration, and HOMA index were more elevated than lean mice, indicating an impairment in glucose metabolism, which was not improved by pistachio consumption (Figure 1D,F). However, regular pistachio intake significantly reduced the HFD-increased serum levels of triglycerides and cholesterol (Figure 1G). In addition, we observed significantly higher ROS and peroxidation lipid levels in HFD plasma than lean. These increases were attenuated in HFD-P plasma (Figure 1H,I).

### 3.2. Effects of Pistachio Intake on Neurodegeneration

**Figure 2:**
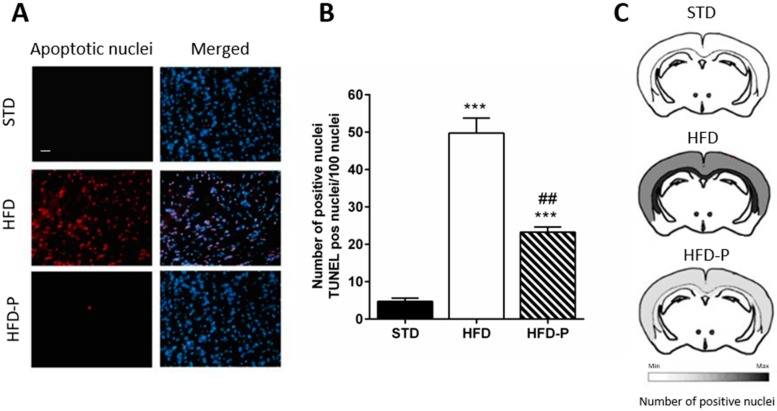
Pistachio consumption exerts a neuroprotective effect. (**A**) TUNEL assay on cerebral cortex sections of STD, HFD, and HFD-P mice; (**B**) the number of apoptotic nuclei in the cerebral cortex; (**C**) scheme of distribution of positive TUNEL nuclei. Data are means ± SEM (*n* = 8/group). *** *p* < 0.001 vs STD, ## *p* < 0.01 vs HFD. Microscope magnification 20×. Bar: 100 µm.

To understand whether regular pistachio intake can reduce the risk of neurodegeneration, the brain was weighed at the time of the sacrifice. Slight differences in brain weight were observed among the three groups. However, a significant reduction of the brain/body weight ratio was found in the HFD group in comparison with STD. This reduction was less pronounced in HFD-P (Table 1). Moreover, a significantly increased number of fragmented nuclei was found in the cerebral cortex of HFD mice compared to STD and HPD-P mice, suggesting that pistachio consumption can counteract neurodegeneration (Figure 2A–C).

### 3.3. Regular Pistachio Consumption Improves HFD-Induced Lipid Dysmetabolism in the Brain

**Figure 3:**
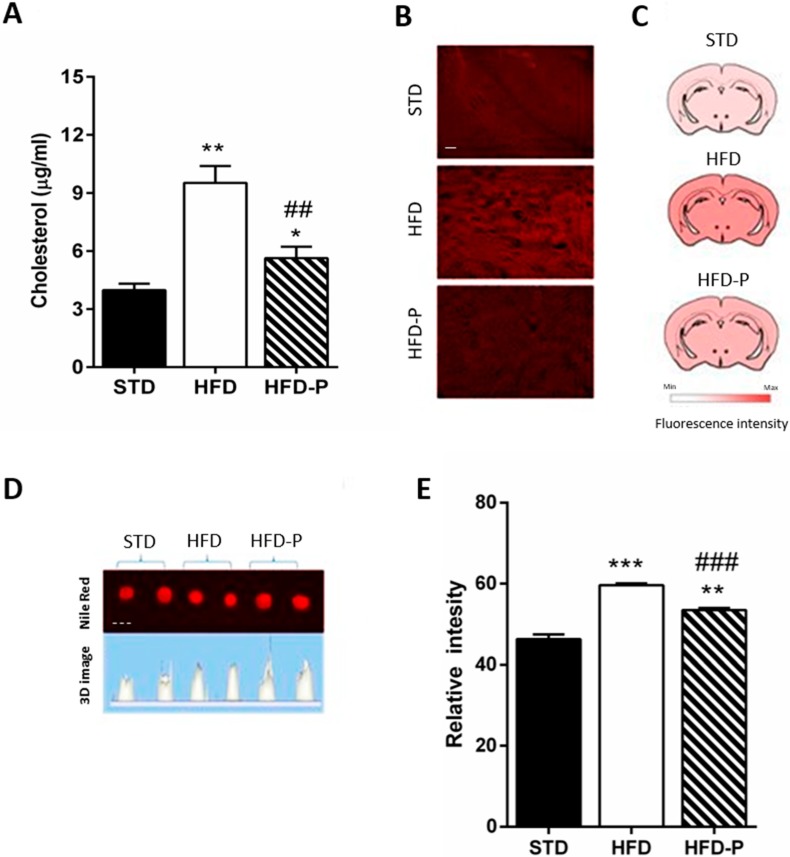
Pistachio consumption prevents HFD-induced brain lipid accumulation. (**A**) cholesterol concentration in brain tissue of STD, HFD, and HFD-P fed mice; (**B**) lipids content measured by Nile Red staining in coronal brain sections of STD, HFD, and HFD-P mice; (**C**) scheme of distribution of fluorescence after Nile Red staining; (**D**) fluorescence in brain lysates of STD, HFD, and HFD-P mice after Nile Red staining; (**E**) quantification of Nile Red staining fluorescence. Data are means ± SEM (*n* = 8/group). * *p* < 0.05, ** *p* < 0.01, *** *p* < 0.001 vs STD. ## *p* < 0.01, ### *p* < 0.001 vs HFD. Microscope magnification 20×. Continuous line, Bar: 100 µm. Dashed line, Bar: 5 mm.

Since obesity and aberration of lipid homeostasis are often linked to neurodegenerative disorders [23,24], we analyzed the brain cholesterol and lipid content in the different animal groups. Significantly higher levels of cholesterol were found in the HFD brain in comparison with STD and HFD-P brain (Figure 3A). Furthermore, large and homogeneous distribution of lipids in HFD coronal sections was observed, while in the HFD-P brain, it was less and more similar to the STD brain (Figure 3B,C). To quantify the cerebral lipids, the whole brain homogenate was stained with Nile Red. The fluorescence intensity, which was significantly increased in the HFD brain, was less in the HFD-P brain (Figure 3D,E).

### 3.4. Pistachio Reduces Oxidative Stress in the Brain of HFD Mice

**Figure 4:**
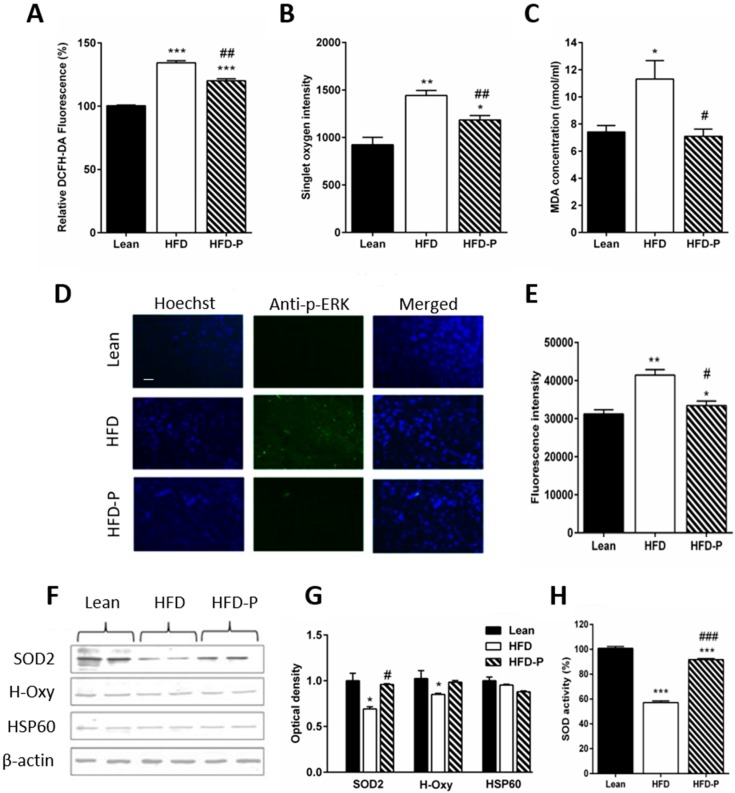
Pistachio consumption prevents HFD-induced brain oxidative stress. (**A**) levels of ROS in brain tissue of lean, HFD or HFD-P fed mice measured by DCFH-DA assay (% respect to STD); (**B**) ^1^O_2_ intensity; (**C**) lipid peroxidation levels in brain tissues of lean HFD or HFD-P fed mice measured by MDA assay; (**D**) immunofluorescence of cerebral cortex sections of lean, HFD, and HFD-P mice incubated with anti-phosho-ERK; (**E**) quantification of p-ERK immunofluorescence; (**F**) Western blot of proteins extracted from lean, HFD, HFD-P brain lysates, and incubated with anti-SOD2, anti-H-Oxy, and anti-HSP60. Uniformity of gel loading was confirmed by β-actin as standard; (**G**) densitometric analysis of immunoreactivity; (**H**) total SOD2 activity levels expressed as % respect to lean in each tissue extract. Data are means ± SEM (*n* = 8/group). * *p* < 0.05, ** *p* < 0.01, *** *p* < 0.001 vs STD. (# *p* < 0.05, ## *p* < 0.01, ### *p* < 0.001 vs HFD. Microscope magnification 20×. Bar: 100 µm.

Lipid dysregulation is linked to brain metabolic stress, which is a risk factor for the neurodegeneration [2], for this reason, we verified the stress conditions in the brain. By ROS and ^1^O_2_ assays, we observed high levels of ROS in the brain of HFD mice that were partially counteracted by a diet with pistachios (Figure 4A,B). Accordingly, reduced lipid peroxidation was found in the brain of HFD-P mice (Figure 4C). Furthermore, the immunoreactivity of p-ERK, a typical stress marker, was found exclusively in sections of the HFD cerebral cortex and not in other brain regions such as the hippocampus, thalamus, and hypothalamus.

We also observed a less immunoreactivity of p-ERK in the HFD-P group (Figure 4D,E) and downregulation of SOD2 and H-Oxy in the brain of HFD mice (Figure 4F,G). In contrast, HFD-P mice showed a level of expression similar to lean for both proteins (Figure 4F,G). No significant difference in HSP60 levels of expression was observed. Instead, decreased SOD2 activity was found in the HFD group as compared to the lean or HFD-P group (Figure 4H).

### 3.5. Pistachio Regular Intake Maintains Mitochondrial Homeostasis

**Figure 5:**
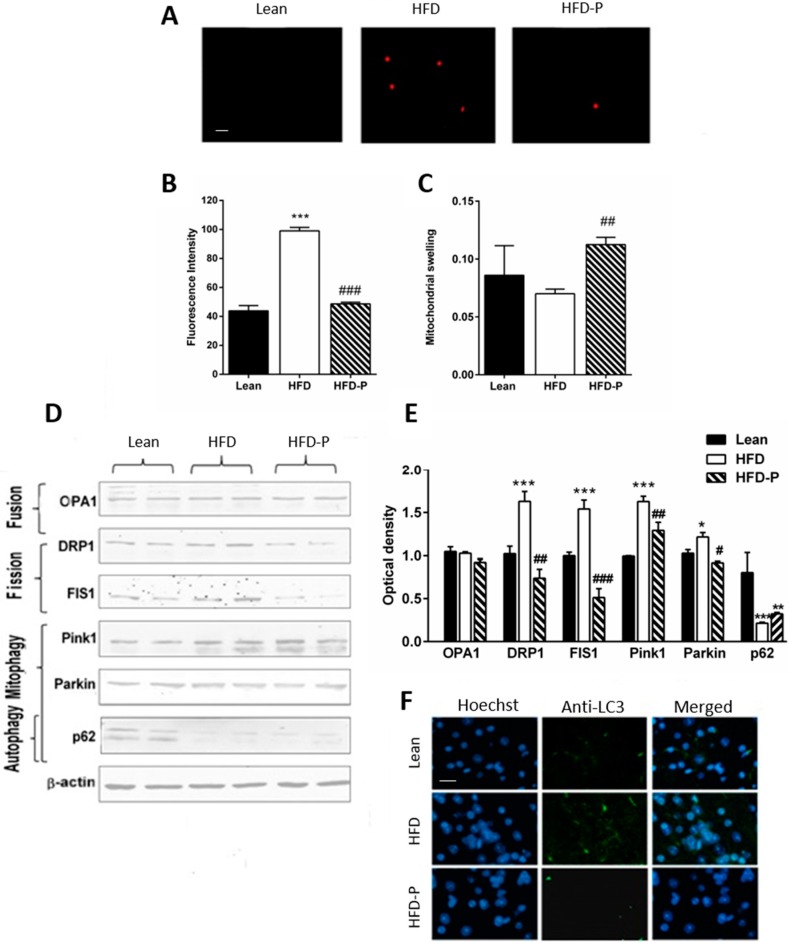
Pistachio intake counteracts HFD-induced mitochondrial dysfunction. (**A**) mitochondrial stress in enriched mitochondria fraction from lean, HFD, or HFD-P fed mice by MitoSox staining; (**B**) level of fluorescence intensity by MitoSox assay; (**C**) mitochondria swelling in lean, HFD, and HFD-P brains; (**D**) Western blot of proteins extracted from brains of lean, HFD, or HFD-P mice and incubated with antibodies against proteins involved in mitochondrial dynamics (OPA1, DRP1, and FIS1), and mitophagy (PINk1, Parkin, and p62). Uniformity of gel loading was confirmed with β-actin as standard. (**E**) densitometric analysis; (**F**) immunofluorescence of cerebral cortex sections of lean, HFD, or HFD-P mice incubated with anti-LC3. Data are the means ± SEM (*n* = 8/group). * *p* < 0.05, ** *p* < 0.01, *** *p* < 0.001 vs STD. # *p* < 0.05, ## *p* < 0.01, ### *p* < 0.001 vs HFD. Microscope magnification 40×. Bar: 50 µm.

Mitochondrial dysfunction is a consequence of oxidative stress, and it is caused by different factors, including impairment of the dynamics. After mitochondria separation, MitoSox assay was used to identify mitochondrial superoxide selectively, and the swelling test was performed as an indicator of permeability transition pore (PTP). High levels of oxidative species inside the mitochondria and increased swelling were found in HFD mice. These conditions were attenuated by a diet, including pistachio (Figure 5A–C). Furthermore, expression of proteins involved in fission and fusion events and ubiquitin-dependent mitophagy were analyzed. No significant difference in OPA1, a protein involved in the fusion process, was observed among the three different groups. In contrast, DRP1 and FIS1, proteins involved in the fission process, were more expressed in the HFD group, than HFD-P fed mice. In the HFD brain, the expression of Pink1, a mitochondrial damage sensor, and Parkin, a signal amplifier, was increased. In contrast, the expression of p62, the signal effector, was decreased, suggesting the presence of damaged mitochondria, a condition partially recovered by HFD-P (Figure 5D,E). Finally, the accumulation of LC3 in HFD coronal brain sections was observed by immunofluorescence assay, confirming the increase of mitophagy and lysosomal activity compared to lean. At the same time, LC3 was less expressed in the HFD-P samples (Figure 5F).

## 4. Discussion

The present study provides experimental evidence for the beneficial neuroprotective effects of regular pistachio intake in the brain of obese mice. This preventive action takes place through the reduction of lipid dysmetabolism, oxidative stress, and mitochondrial dysfunction.

It is now widely accepted that the consumption of HFD is a risk factor for the development of obesity-related diseases and prolonged HFD feeding has been reported to accelerate the pathogenesis of neurodegeneration [5,28,29], leading to impairment of cognitive functions in rodents [29].

Although an increasing number of experimental and clinical observations have led to consider almonds, hazelnuts, and walnut as brain-protective agents, mainly against brain atrophy, memory loss, and Alzheimer’s disease [30], few studies can be found about pistachio. Indeed, Singh et al. [31] demonstrated that *Pistacia vera* fruit extracts improve mouse cognitive processes after chemically-induced deficits; however, the mechanisms responsible have not been clarified yet. Pistachio regular consumption has already been shown to enhance the obesity-related dysfunctions, including inflammation, by positively modulating the expression of genes linked to the lipid metabolism [4,15]. Therefore, the goal of the present study was to verify whether supplementing an HFD with pistachio fruits can mitigate the harmful effects associated with the consumption of an HFD in the brain of obese mice.

The potential beneficial effect of pistachio on neurodegeneration was investigated by using an HFD fed mouse that provided a suitable model for studies on diet-induced metabolic changes and redox equilibrium disorders [5]. First, we determined if supplementation of an HFD with pistachio affected the neurodegeneration already showed to be present in the mouse cerebral cortex after 8 weeks on HFD [32]. We found a neuroprotective action of pistachio consumption, which partly prevented HFD-induced neuronal apoptosis, as demonstrated by a reduced number of cells with fragmented DNA in the cortical areas. Indeed, as we previously reported, the brain dysfunctions in long term HFD fed mice are associated with peripheral and central insulin resistance [5] and dyslipidemia [7]. For this reason, we verified the hypothesis that regular pistachio intake could influence glucose or lipid metabolism. The results obtained by measuring the metabolic parameters allow us to discard the supposition that the effects of pistachio consumption in the brain are a direct consequence of actions on glucose metabolism or bodyweight, because no changes in fasting glycemia, HOMA index, or body weight were observed. It was noted that the inability of chronic pistachio intake to change the bodyweight or influencing glucose dysmetabolism was already reported both in humans and animal models [15,33]. Our results suggest that the effects of regular pistachio intake are attributable to beneficial actions on lipid dysmetabolism and redox state. This is in agreement with previous studies on animals [15,34,35] and humans [36,37], where pistachio intake was able to decrease the HFD-induced high levels of plasma cholesterol, triglycerides, and oxidative stress, suggesting lipid-lowering and antioxidant properties of pistachio fruit. Thus, we cannot exclude that the antioxidant content of pistachio can activate a compensatory mechanism for mainly mitigating HFD-induced systemic and central dysmetabolism and redox stress.

An altered lipid metabolism is believed to be a critical event that contributes to central nervous system injuries [38]. In our experiments, HFD induced high cholesterol and neutral lipid levels in the brain. Hyperlipidemia and more exactly high cholesterol levels have been reported in the brains of patients with Alzheimer’s disease [26], and they have been shown to worsen brain injury in an experimental mouse model [39]. A recent study reported that increased free cholesterol induces neuronal death via endoplasmic reticulum stress and activation of apoptotic mechanisms [40]. Noteworthy, the pistachio anti-lipotoxic effect was also found in the brain, as evidenced by the reduction in lipids and oxidative stress, suggesting a beneficial protective action against neuronal damage. Of note is that the oxidative lipids and ceramides, increased by systemic dysmetabolism, are able to pass through the blood-brain barrier, and they can also contribute to the oxidative dysmetabolism that is already occurring in the brain, accelerating the progression of the neurodegeneration [7,41]. In agreement with these findings, our results indicate that the antioxidant activity of pistachio improves dysfunctional status and reduces the HFD-induced redox stress. Oxidative stress plays a crucial role in various types of nervous system damage, including neurodegenerative disorders [42]. Indeed, the cerebral tissue is among the most vulnerable tissues to oxidative stress because of its high levels of polyunsaturated fatty acids that are susceptible to lipid peroxidation. Moreover, it also has lower antioxidant defenses in comparison with other organs [43,44]. Fatty acid oxidation produces ROS, which can induce neurodegeneration via apoptotic pathways [45,46]. The HFD model is clearly lipotoxic due to the high ROS and lipoperoxidation levels, the increased expression of p-ERK, and down-regulation of H-Oxy and SOD2 that we found in HFD brains.

In agreement with previous reports [47], the activity of the SOD2 was lower in the HFD brain, suggesting that reduced antioxidant defense may contribute to the increased oxidative stress. However, the changes in the stress markers were less pronounced in the HFD-P brain, confirming the antioxidant activity of the pistachio, already demonstrated in other HFD tissues [15]. These properties could be related to the presence of phytosterols (stigmasterol and campesterol), lutein (xanthophyll carotenoid), and polyphenols (resveratrol and catechins) [48,49].

Impaired mitochondrial function is one factor that contributes to degenerative brain disorders [50], and the mitochondrial dysfunction has been shown to be the primary cause of the cellular apoptosis [51]. We found significantly increased brain mitochondrial stress and swelling after long-term HFD, indicative of mitochondrial dysfunction [52]. Mitochondrial swelling is the endpoint of a cascade of events induced by excessive ROS generation and including altered Ca^2+^ uptake and PTP opening. A balance between fission and fusion of mitochondria is critical for mitochondrial functional integrity, and an increase of fission events can produce accumulation of damaged mitochondria that can be removed by selective mitophagy. In our experimental conditions, HFD disrupts the mitochondrial homeostasis, as demonstrated by overexpression of DRP1 and FIS1, proteins involved in the fission process, and by the changes in the expression of Pink1, parkin, and p62, proteins involved in the pathway that regulates ubiquitin-dependent mitophagy. Moreover, HFD induces autophagy-related processes, as suggested by LC3 increased expression.

Interestingly, regular pistachio intake seems to counteract these adverse effects of HFD by preventing the mitochondrial brain dysfunction and autophagosome-lysosome fusion, suggesting once more the beneficial effects of pistachio fruit consumption on brain health. In agreement with our results, several findings indicate that antioxidants of pistachio fruit, including phytosterols and polyphenols, influence mitochondrial dynamics by maintaining the balance between fusion and fission and preventing mitophagy. In addition, modification of autophagy by polyphenols has been proposed as a promising therapeutic strategy [53].

Further, we have to consider that selective mitophagy leads to mitochondrion number reduction with a consequent decrease in ATP production, as well as reduced metabolic activity in the brain. Preclinical and clinical studies have demonstrated that a diet supplemented with antioxidants and combined with exercise training can stimulate mitochondrial biogenesis, a mechanism that can replace the loss of damaged mitochondria [54,55]. Thus, a diet, including pistachio, especially in combination with exercise training, can represent an efficient strategy to improve dysmetabolism and delay neurodegeneration.

## 5. Conclusions

**Figure 6:**
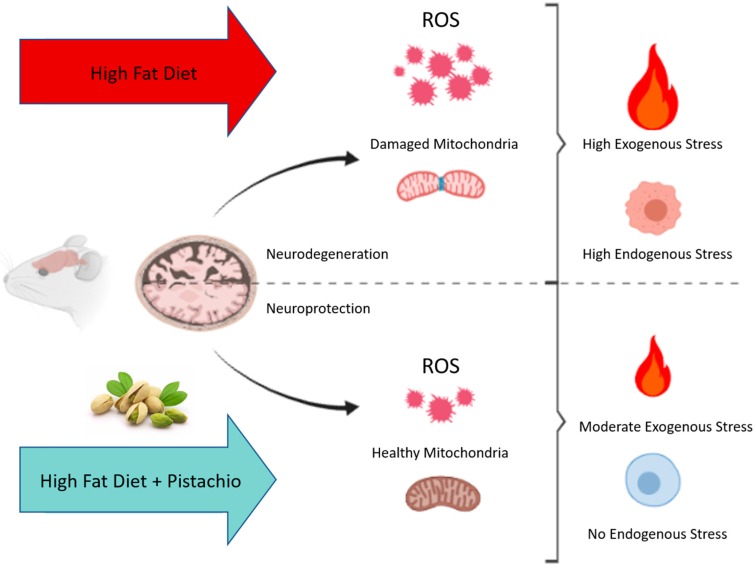
Schematic representation of the neuroprotective effect of regular pistachio intake.

In conclusion, our results demonstrate that pistachio consumption has beneficial effects against the negative impact induced by long-term HFD in the mouse brain by exerting neuroprotective activities. The neuroprotective effects include decreased brain apoptosis, decreased brain lipid, and oxidative stress with the associated improvement of mitochondrial function. In particular, regular pistachio intake attenuates mitochondrial ROS generation induced by a high-fat diet, which in turn reduces damaged mitochondria (Figure 6), leading to beneficial effects on mitochondrial dynamics and mitophagy.

## Figures and Tables

**Table 1:** Effects of pistachio diet on body weight and brain weight. HFD-P showed significantly increased brain/body weight ratio in comparison with HFD, suggesting a preventive action of pistachio consumption against brain atrophy.

Diet	Mouse C57BL/6	Age (Months)	Body Weight (g) (±SEM)	*p*-Value	Brain Weight (g) (±SEM)	*p*-Value	Weight Ratio Brain/Body	*p*-Value
STD	8	4	30.4 ± 0.09		0.31 ± 0.09		0.01019 ± 0.002	
HFD	8	4	44.2 ± 0.02 *	<0.005	0.28 ± 0.06 *	<0.05	0.00633 ± 0.001 *	<0.05
HFD-P	8	4	42.1 ± 2 *		0.30 ± 0.05 *	<0.05	0.00712 ± 0.002 ^#^	<0.05

STD, standard diet, HFD, high-fat diet; HFD-P, a high-fat diet supplemented with pistachio; SEM, standard error medium; * denotes significant difference compared with the STD; ^#^ denotes significant difference compared with the HFD group.
